# Selectivities of Carbon Dioxide over Ethane in Three Methylimidazolium-Based Ionic Liquids: Experimental Data and Modeling

**DOI:** 10.3390/molecules29174152

**Published:** 2024-09-01

**Authors:** Nadir Henni, Amr Henni, Hussameldin Ibrahim

**Affiliations:** Clean Energy Technologies Research Institute (CETRi), University of Regina, Regina, SK S4S 0A2, Canada; nadir.henni1@gmail.com

**Keywords:** ionic liquid, ethane, carbon dioxide, solubility, selectivity, equation of state

## Abstract

This work focused on the solubility of ethane in three promising ionic liquids {1-Hexyl-3-methylimidazolium bis(trifluormethylsulfonyl) imide [HMIM][Tf2N], 1-Butyl-3-methyl-imidazolium dimethyl-phosphate [BMIM][DMP], and 1-Propyl-3-methylimidazolium bis(trifluoromethyl-sulfonyl)-imide [PMIM][Tf2N]}. The solubilities were measured at 303.15 K to 343.15 K and pressures up to 1.4 MPa using a gravimetric microbalance. The overall ranking of ethane solubility in the ionic liquids from highest to lowest is the following: [HMIM][Tf2N] > [PMIM][Tf2N] > [BMIM][DMP]. The Peng–Robinson equation of state was used to model the experimental data using three different mixing rules: van der Waals one, van der Waals two, and Wong–Sandler mixing rules combined with the Non-Random Two-Liquid model. The average absolute deviations for the three mixing rules for the ionic liquids at the three temperatures were 4.39, 2.45, and 2.45%, respectively. Henry’s Law constants for ethane in [BMIM] [DMP] were the highest (lowest solubility) amongst other ionic liquids studied in this work. The solubility ranking for the 3 ILs was confirmed by calculating their overall polarity parameter (N) using COSMO-RS. The selectivity of CO_2_ over C_2_H_6_ was estimated at three temperatures, and the overall ranking of the selectivity was in the following order: [PMIM][Tf2N] > [BMIM][DMP] > [HMIM][Tf2N] > Selexol. Selexol is an efficient and widely used physical solvent in gas sweetening. It has lower selectivity than the three ionic liquids studied. [PMIM][Tf2N], a promising solvent, has the highest selectivity among the three ILs studied and would, therefore, be the best choice if, in addition to carbon dioxide capture, ethane co-absorption was to be avoided. The enthalpy and entropy of solvation at infinite dilution were also estimated.

## 1. Introduction

As reported by the Energy Information Agency (EIA) in 2023, approximately 83% of the total energy produced in the United States came from fossil fuels, i.e., oil, coal, and natural gas [1]. Fossil fuels, when burned, produce large quantities of CO_2_ and other gases. These gases will result in greenhouse gas effects, resulting in the absorption of the heat coming from the sun and causing the temperature of the earth’s atmosphere to rise. Global warming is consequently considered a detrimental and even catastrophic change in the climate. According to the Scripps Institution of Oceanography and the National Oceanic and Atmospheric Administration (NOAA), the CO_2_ May monthly average in 2024 reached 426.7 ppm, an increase of 2.92 ppm over that recorded in 2023 [2]. NOAA also reports that 2023 is the warmest year on record by far [3].

There are multiple ways of slowing down the rate of global warming. One of those methods is through emission reduction with carbon capture. The most up-to-date technology in carbon capture is the process of absorbing CO_2_ generated by power generation, steel, and cement plants. This latest technology is similar to the removal of acid gases from natural gas sweetening, except that a mixture of amines is used as a solvent. Capturing CO_2_ using non-aqueous solvents is an advanced, next-generation post-combustion capture technology that seeks to remove CO_2_ emitted from power generation and industrial flue gas streams.

Natural gas is one of the types of energy from the diverse portfolio of fossil fuels. It is a naturally occurring hydrocarbon composed mainly of methane. In general, it also contains ethane, propane, butane, and pentane, as well as non-hydrocarbon gases such as nitrogen, carbon dioxide, and hydrogen sulfide. Natural gas is formed from the remains of plants and animals exposed to extreme heat and pressure under the earth’s surface hundreds of millions of years ago. It constitutes the major source of energy consumed today; the demand for natural gas has not ceased to increase year after year in the last decade. According to the International Energy Agency, the world’s natural gas consumption reached 4138 billion cubic meters (bcm) in 2022 and is expected to reach 4299 bcm in 2050 [4].

To this day, carbon capture is performed by capturing CO_2_ from both high-pressure natural gas streams and low-pressure gas streams with amines-based chemical solvents [5]. However, this process is not very economical in the case of flue gases because of the large amount of energy required to regenerate the solvent and the serious solvent degradation that occurs. For natural gas applications, using physical rather than chemical solvents for carbon capture is more economical when the cleaning targets are not at the ppm level. Multiple processes have been used for carbon capture depending on the partial pressure of CO_2_ in the feed and the required degree of treatment. Aside from amines and their blends, organic physical solvents are one of these processes. One of the main advantages is the low energy needed for solvent regeneration [5]. Commonly, physical solvents have a low energy of regeneration and relatively low vapor pressure. They are non-corrosive, with high thermal stability. They could be used in treating natural gas as standalone or mixed with aqueous amines in the case of flue gases. Ionic liquids (ILs) represent a category of physical solvents that utilize intermolecular forces for CO_2_ capture. ILs are normally defined as compounds composed of ions (a cation and an anion) with a melting point below 100 °C. In this study, three new and promising ionic liquids are studied to analyze their capacities for ethane at three different temperatures (303.15 K, 323.15 K, and 343.15 K). This range of temperatures is of great importance to the industry and the pressures used were up to 1.4 MPa. Using CO_2_ solubility data, we recently published, selectivity values are reported for the three solvents at three temperatures. The experiments were conducted using a gravimetric microbalance (IGA-003).

## 2. Results

### 2.1. Verification of the C_2_H_6_ Solubility Measurement

**Figure 1:**
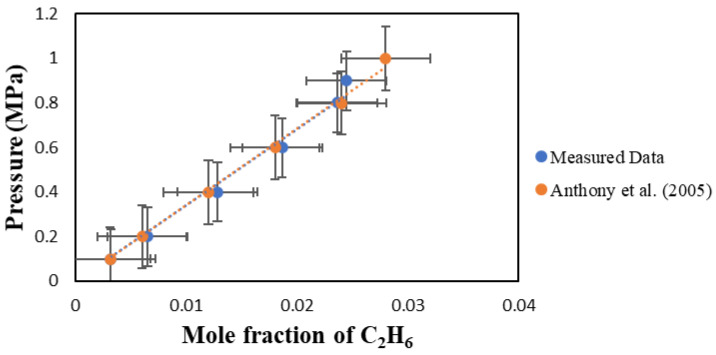
Comparison between the measured data for the solubility of C_2_H_6_ in [BMIM][PF6] and those reported by Anthony et al. (2005) [6] at 298.15 K.

A verification test was performed to ensure the IGA-003 is operating as it should. In this verification test, [BMIM][PF6] was used as the solvent, and the results were compared with Anthony et al. (2005) at 298.15 K [6]. As shown in Figure 1, the results between the measured data and those published by Anthony et al. are in excellent agreement.

### 2.2. Solubility of C_2_H_6_ in Ionic Liquids

**Figure 2:**
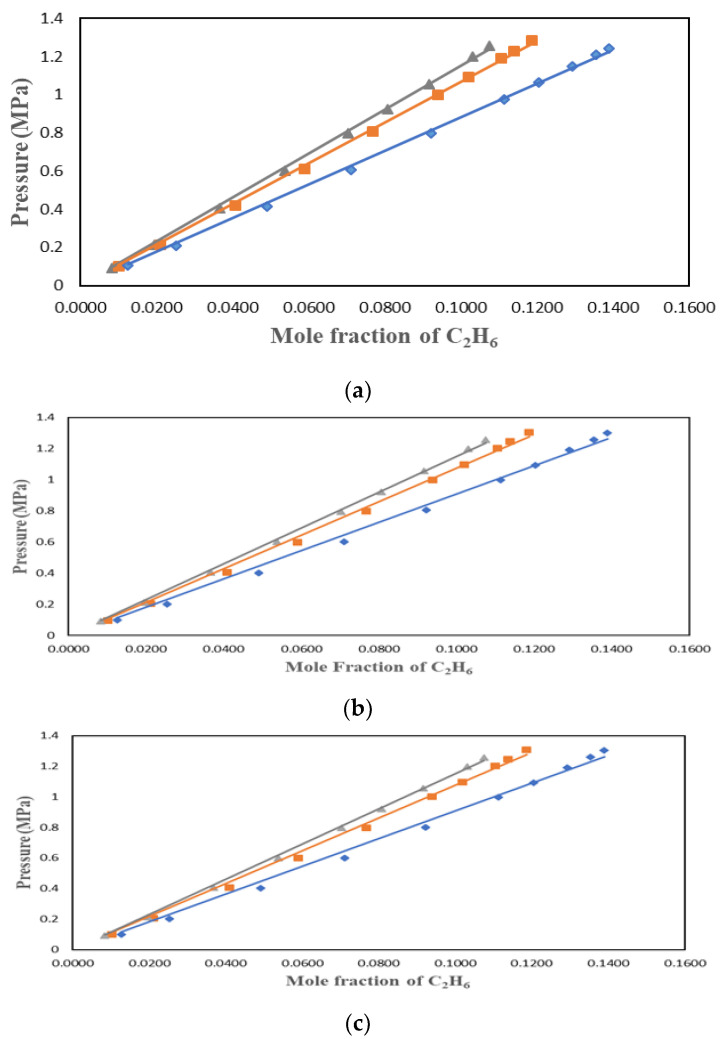
Solubility data for C_2_H_6_ in [HMIM][Tf2N] at different temperatures and pressures up to 1.4 MPa: ♦ 303.15 K; ■ 323.15 K; ▲: 343.15 K. Experimental VLE data were correlated with (**a**) PR + vdW1, (**b**) PR + vdW2, and (**c**) PR + WS + NRTL.

**Figure 3:**
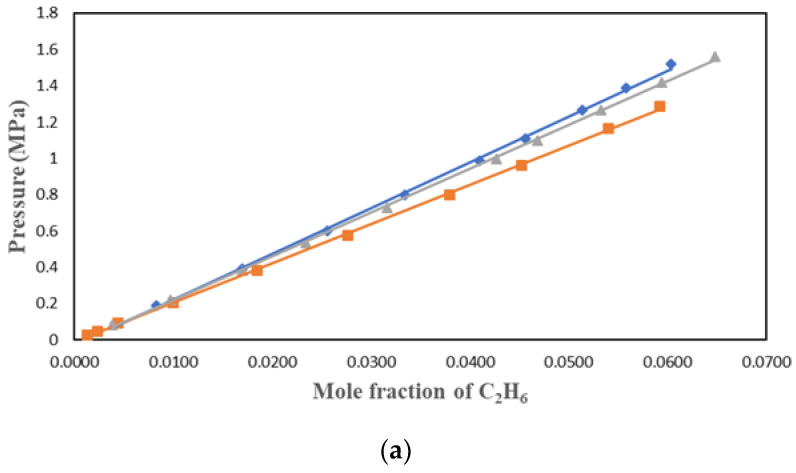

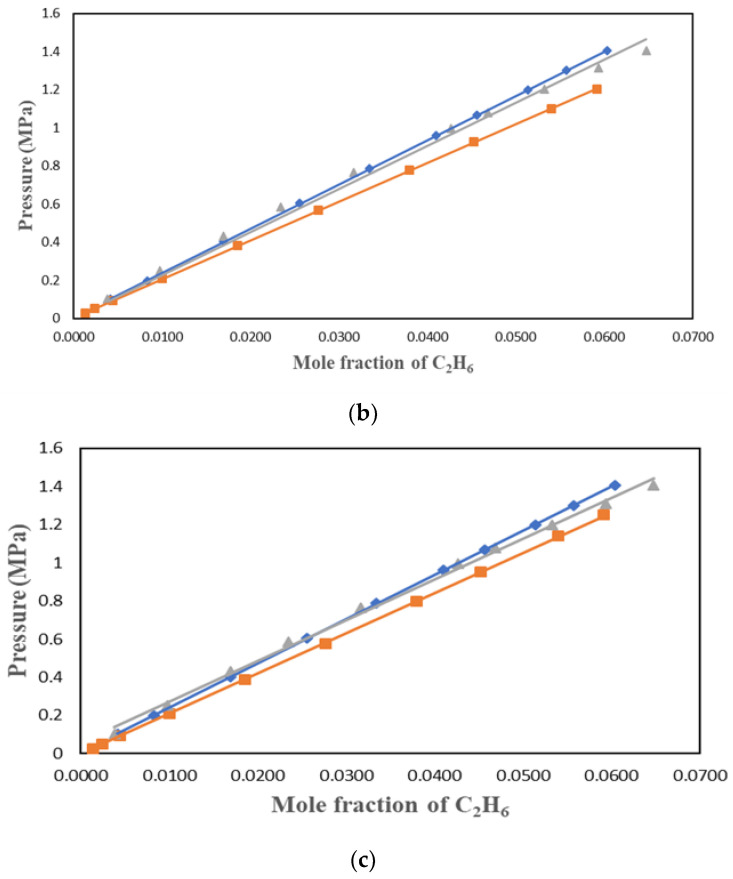
Solubility data of C_2_H_6_ in [BMIM][DMP] at different temperatures: ♦ 303.15 K; ■ 323.15 K; ▲ 343.15 K. Experimental VLE data were correlated with (**a**) PR + vdW1, (**b**) PR + vdW2, and (**c**) PR + WS + NRTL.

**Figure 4:**
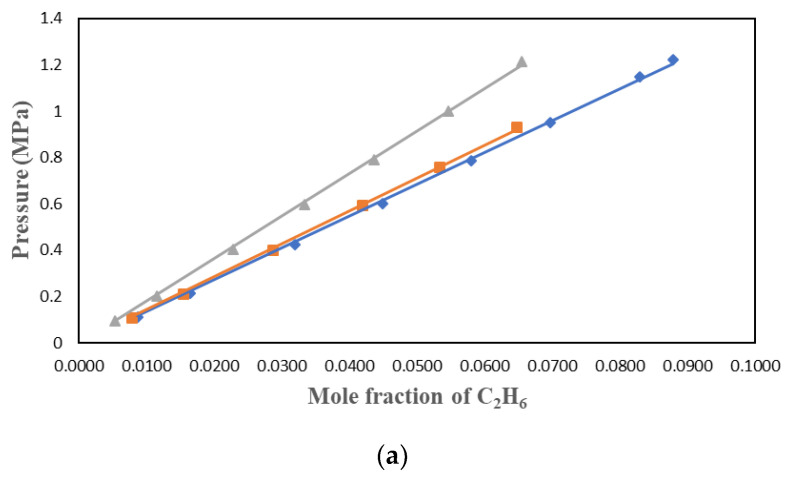

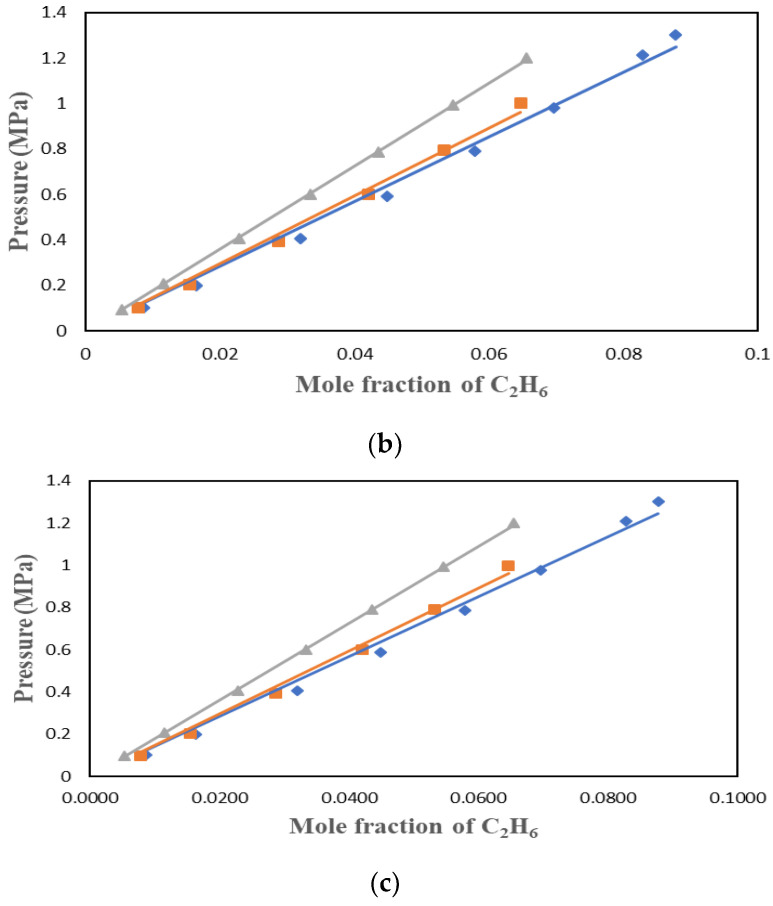
Solubility data of C_2_H_6_ in [PMIM][Tf2N] at different temperatures: ♦ 303.15 K; ■ 323.15 K; ▲ 343.15 K and model correlation: lines with (**a**) PR + vdW1, (**b**) PR + vdW2, and (**c**) PR + WS + NRTL.

C_2_H_6_ solubility data were studied by many researchers who found the ionic liquids to have lower solubility than CO_2_. This work used three ionic liquids with different cations and anions to understand the factors affecting the solubility of C_2_H_6_ in ILs. C_2_H_6_ solubility in [HMIM][Tf2N], [BMIM][DMP], and [PMIM][Tf2N] were measured at 303.15 K, 323.15 K, and 343.15 K and pressures up to 1.4 MPa. The results were plotted in P-*x* form in Figure 2, Figure 3 and Figure 4 for the three solvents. The values of the experimental data can be found in Table 1, Table 2 and Table 3.

## 3. Discussion

### 3.1. Factors That Affect the Solubility of C_2_H_6_

#### 3.1.1. Pressure and Temperature

Figure 2, Figure 3 and Figure 4 show that temperature and pressure affect the solubility of C_2_H_6_ in ionic liquids. As expected, it is observed that as the temperature rises, C_2_H_6_ solubility in the ionic liquid decreases. As pressure rises, C_2_H_6_ solubility increases.

**Figure 5:**
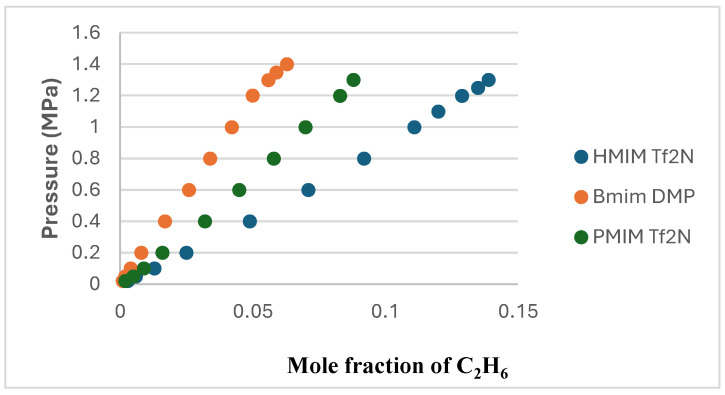
Comparison between the solubility of C_2_H_6_ in the 3 ILs at 303.15 K.

As presented in Figure 5, a comparison between the solubility of ethane in the three ionic liquids at 303.15 K shows that the solvent that absorbs ethane the least is [BMIM][DMP].

#### 3.1.2. Anion Effect

A significant amount of research has been performed to understand the mechanism of how different ionic liquids have different solubilities when it comes to capturing gases such as CO_2_, CH_4,_ C_2_H_6,_ and others. Anthony et al. [6] studied the anion effect on CO_2_ solubility in [BMIM]-based ionic liquids with three different anions. Experiments were performed between 283.15K and 323.15 K and up to 1.4 MPa for three ionic liquids with three different anions, namely [BMIM][Tf2N], [BMIM][BF4], and [BMIM][PF6]. They reported that ethane, along with other gases, such as ethylene, oxygen, and argon, have the lowest solubilities in ILs compared to other common solvents.

#### 3.1.3. Alkyl Chain Length with [Tf2N]-Anion

**Figure 6:**
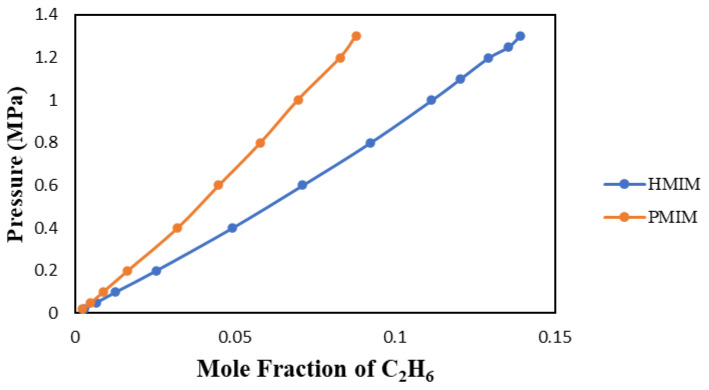
Solubility of C_2_H_6_ in the two ILs with [Tf2N]-anion at 303.15 K.

As presented in Figure 6, the alkyl chain effect will affect the solubility of C_2_H_6_ in ionic liquids. [HMIM] and [PMIM] are attached to an imidazolium-based cation with an n-methyl chain. The difference between their molecular structures is due to the hexyl and propyl alkyl chains. In their carbon chains, hexyl has six methyl groups, and propyl has three. The longer the chain, the more gas the ionic liquid will absorb. This observation was made also by Zoubeik (2014) [7] and Tagiuri et al. (2014) [8] based on several studies published in the literature. Note that the polarity parameter (N) for [PMIM] is lower than that of [HMIM], confirming its higher capacity in absorbing ethane. The polarity parameter is discussed further at the end of Section 3.1.4 and also in reference [9].

#### 3.1.4. Cation with the Same [Tf2N] Anion

The effect of different cations with the same [Tf2N] anion is observed in Figure 6. The [PMIM] cation allowed for less C_2_H_6_ solubility than the [HMIM] cation. The solubility in ILs with the same anions will depend mostly on the cation. Based on studies published in the literature, methyl imidazolium cation-based ionic liquid tends to usually have a higher C_2_H_6_ solubility than other cation-based ionic liquids. The solubility of C_2_H_6_ in [HMIM] is higher than that in [PMIM], most probably because of its longer carbon (alkyl) chain, as discussed earlier in the section entitled “Alkyl Chain Lengths”. As reported by Taguiri (2019) [10] and others, this study confirms the fact that the anion of an ionic liquid has more of an impact on gas solubility than its cation.

For detailed information about the mechanism of gas solubility in ionic liquids, please refer to a comprehensive screening study we performed in 2011 [9] using COSMO-RS. In it, molar volumes and polarity of the ionic liquids were used to quantify the solubility of gases in ILs. The study dealt with gases such as CO_2_ and the selectivity of methane and nitrogen. A relative overall polarity parameter N was defined and computed for 2701 ionic liquids. N quantifies the degree of chemical affinity of water with ions due to vdW, misfit, and H-bond. The more negative the value of N, the more polar the IL is. N was found to be equal to 6.22 for [Tf2N], −62.04 for [DMP], 37.16 for [HMIM], 32.79 for [BMIM], and 32.70 for a cation similar to [PMIM]. Accordingly, [BMIM][DMP] with the lowest N should have the lowest ethane solubility, followed by [PMIM][Tf2N] and [HMIM][Tf2N]. This predicted trend is confirmed by the experimental data presented in Figure 5. A comprehensive description of the structural variations of the ILs on the solubility can also be found. In addition to the alkyl chain, alkylation of ammonium and phosphonium cation, change in cation family for ring cation and non-ring cation, and presence of hydroxyl and ether groups were discussed in reference [9].

### 3.2. Optimization of Binary Interaction Parameters

MATLAB was used for the thermodynamic model and was based on a bubble point technique for the three mixing rules [5]. As shown in Equation (1) [10], the binary interaction parameters were optimized by minimizing the objective function (Err). Because experimental data at lower pressures are more likely to be erroneous, the binary interaction parameters were optimized using a pressure range of 0.1 to 1.4 MPa.

(1) $$Err=\frac{100}{n}\sum_{i=1}^{n}\left[\frac{P_{Exp,i}-P_{Cal,i}}{P_{Exp,i}}\right]$$

The average absolute deviation for the vdW1, vdW2, and WS-NRTL mixing rules when used in the ionic liquids for C_2_H_6_ were 4.39%, 2.45%, and 2.45%, respectively. The results for C_2_H_6_ were best when using either the vdW2 or WS-NRTL mixing rule, as shown in Table 4.

### 3.3. Henry’s Law Constants, Enthalpies, and Entropies

Using the solubility data, Henry’s Law constants were derived for the three ionic liquids. For each temperature, the fugacities of C_2_H_6_ were plotted against mole fractions. The limiting slope of the data’s second-order polynomial was used to determine Henry’s Law constants for each temperature. Henry’s Law constants $,H_{i},$ of the solute (i) in the solvent (j) is the ratio of the fugacity of the solute (i) to the mole fraction of the solute (i) in solvent (j) at infinite dilution at temperature T and pressure P; $f_{i}^{L}$ and $f_{i}^{V}$ are the fugacity of solute (i) in the liquid phase and vapor phase, respectively; and $x_{i}$ and $y_{i}$ are the mole fraction of solute (i) in the liquid phase and vapor phase, respectively, at temperature T and pressure P [5]. It is expressed as the following:

(2) $$H_{i}=\underset{x_{i}\to 0}{\lim}\frac{f_{i}^{L}\left(T,P,x_{i}\right)}{x_{i}}=\underset{x_{i}\to 0}{\lim}\frac{f_{i}^{V}\left(T,P,y_{i}\right)}{x_{i}}$$

Once Henry’s Law has been estimated from the solubility data, estimating the enthalpy of solvation (ΔH^∞^) at infinite dilution and the entropy of solvation (ΔS^∞^) at infinite dilution can also be performed using the following equations:

(3) $$∆H^{\infty }=R{\left(\frac{\partial \left(\ln H\right)}{\partial \left(1/T\right)}\right)}_{P}$$

(4) $$∆S^{\infty }=-R{\left(\frac{\partial \left(\ln H\right)}{\partial \left(\ln T\right)}\right)}_{P}$$

where ΔH^∞^ is the enthalpy of solvation at infinite dilution, (ΔS^∞^) is the entropy of solvation at infinite dilution, R is the universal gas constant, H is Henry’s Law constant, and T is temperature.

**Figure 7:**
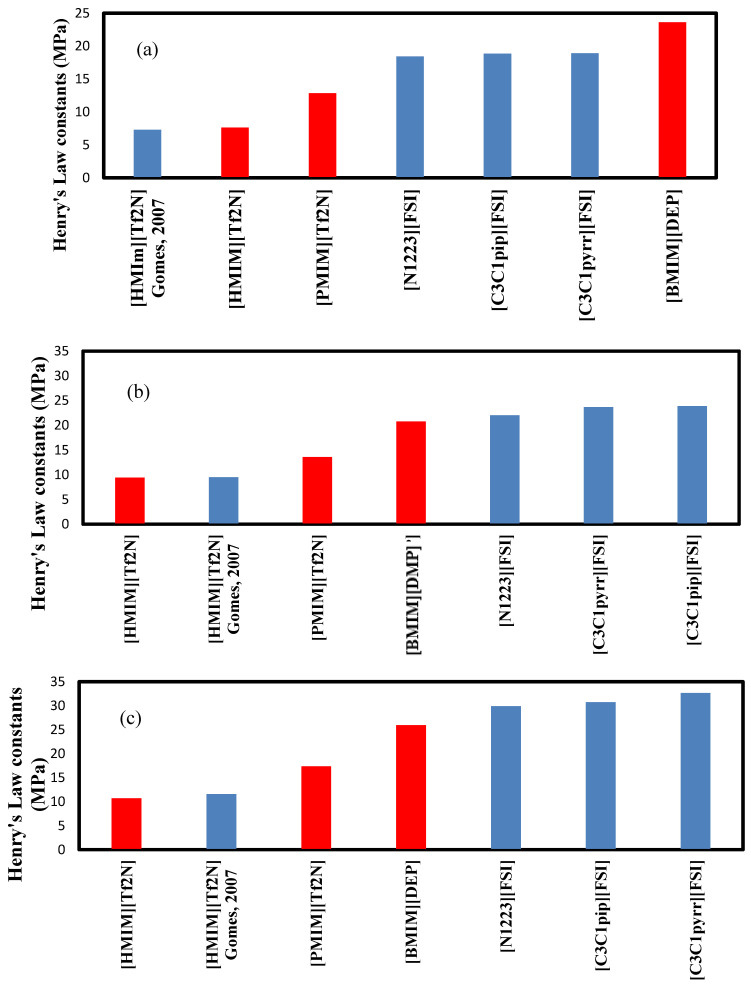
Comparison of Henry’s Law constants for C_2_H_6_ in ILs at (**a**) 303.15 K, (**b**) 323.15 K, and (**c**) 343.15 K; red: ionic liquids used in this work and blue: other ILs obtained from the literature summarized by Nath et al. [12].

The comparison between the three ionic liquids is presented in Figure 7 for the three temperatures studied. Henry’s Law constants for C_2_H_6_ in [HMIM][Tf2N] were published by C. Gomes et al. (2007) [11] and are reported here for comparison. The values are very close, confirming the accuracy of the experimental data. The calculated values are presented in Table 5. Henry’s Law constants of the three ionic liquids studied are compared to those found in the literature and presented in Figure 7 for the three temperatures studied.

Overall, the ranking of the Henry’s Law constants for C_2_H_6_ is the following: [BMIM][DMP] > [PMIM][Tf2N] > [HMIM][Tf2N] at all temperatures. As the highest value is the best, and [BMIM][DMP] is therefore the IL that absorbs ethane the least. Compared to other ionic liquids, the three ionic liquids are not the best, but [BMIM][DMP] seems to be the best option.

The decreasing ranking in terms of the heat of absorption is the following: [BMIM][DMP] > [HMIM][Tf2N] > [PMIM][Tf2N]. The solubility of ethane in three ILs {[C3C1pip][FSI], [C3C1pyrr][FSI], and [N1223][FSI]} reported in Figure 7 was published by Nath et al. (2017) [12].

### 3.4. CO_2_/C_2_H_6_ Selectivity in the Three Ionic Liquid Systems

The major criteria for a potential solvent for the use of gas solubility are the following: high removal efficiency, high selectivity toward CO_2_, and low energy requirement. Not much research has been conducted when it comes to the selectivity between gases. When CO_2_ is captured, it is not the only gas being absorbed. Other gases are absorbed, such as N_2_O, O_2_, SO_2_, H_2_S, CH_4,_ and many other hydrocarbons. Therefore, with that in mind, it is crucial to study the effect of selectivity on ionic liquids. The selectivity of the ionic liquids used in this work was estimated as the ratio between Henry’s Law constant of CO_2_ and C_2_H_6_. The equation used to estimate the selectivity is the following:

(5) $$S_{{CO}_{2}/C_{2}H_{6}}=\frac{H_{C_{2}H_{6}}}{H_{{CO}_{2}}}$$

The values of the selectivities are presented in Table 6. CO_2_/C_2_H_6_ selectivity of the ionic liquids is not significantly high; however, they decrease as the temperature rises. The data obtained for the Henry’s Law constant of CO_2_ was taken from previous research we performed measuring the CO_2_ solubility in [HMIM][Tf2N], [BMIM][DMP], and [PMIM][Tf2N] [13]. The study revealed that the three solvents were among the best in capturing CO_2_. The three ionic liquids followed this rank in terms of increasing CO_2_ solubility: [HMIM] [Tf2N] > [PMIM][Tf2N] >[BMIM][DMP], as reported in reference [13]. The study confirmed that [HMIM][Tf2N] is an excellent solvent for CO_2_ capture. [PMIM][Tf2N] was also found to have a high capacity for CO_2_ among ILs published in the literature.

**Figure 8:**
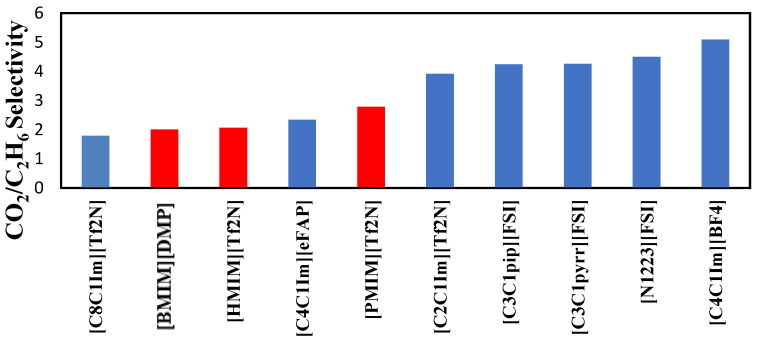
Comparison of CO_2_/C_2_H_6_ selectivity in several ionic liquids reported by Nath et al. [12] at 303.15 K.

**Figure 9:**
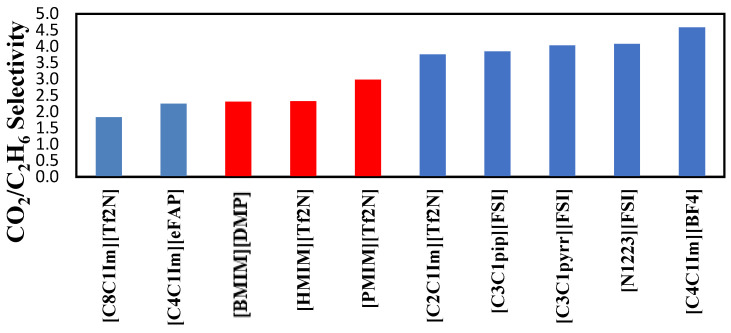
Comparison of CO_2_/C_2_H_6_ selectivity in several ionic liquids reported by Nath et al. [12] at 323.15 K.

**Figure 10:**
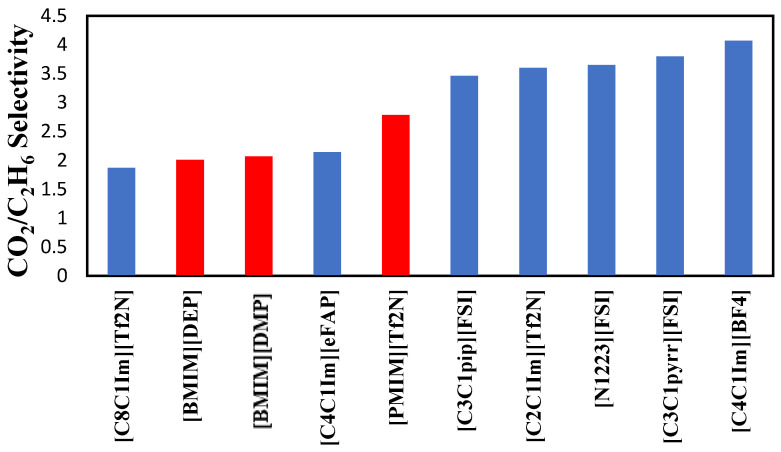
Comparison of CO_2_/C_2_H_6_ selectivity with other ionic liquids reported by Nath et al. [12] at 343.15 K.

The selectivities of the three ionic liquids studied are compared to those found in the literature and presented in Figure 8, Figure 9 and Figure 10 at the three temperatures studied.

For a comprehensive comparison, we have added in the table data related to Selexol, a widely used and efficient physical solvent (a mixture of polyethylene glycol dimethyl ethers). Selectivity values were calculated based on values reported by Rayer et al. (2012) [14].

Overall, the ranking of the CO_2_/C_2_H_6_ selectivity is the following: [PMIM][Tf2N] > [BMIM] [DMP] > [HMIM][Tf2N] > Selexol. As the highest selectivity is the one preferred, among the three ionic liquids studied, [PMIM][Tf2N] is considered the best. As shown below, these ILs do not possess the best selectivities among ionic liquids in the literature. Selexol seems to have the lowest selectivity compared to the ILs selected in this study. More details about the solvents in Figure 8, Figure 9 and Figure 10 can be found in reference [12].

## 4. Materials and Methods

### 4.1. Materials

Table 7 lists the structures, acronyms, suppliers, purities, and water content of all of the solvents and solutes used in this study. The manufacturers of the chemicals provided the information related to the purities of the solvents listed in the same table.

### 4.2. Solubility Measurement

The gas solubility experiments were performed with the use of an IGA-003 (Intelligent Gravimetric Analyzer, Hidden Isochema Ltd., Warrington, UK). The machine measures the weight change of a liquid sample once a gas/gas mixture has been injected into the system. The IGA-003 can run at a maximum pressure and temperature of up to 2.0 MPa and 773.15 K, respectively.

For each isotherm experiment, around 50 to 90 mg of ionic liquid sample was inserted into the sample container to determine the solubility of the gas in the ionic liquid. A Grant water bath was used to regulate the temperature of the sample. For each experiment, the sample was degassed for around 10 h at high temperatures and under a deep vacuum before beginning the isotherm process to remove any contaminants that may have been in the sample. Throughout the degassing process, the temperature of the sample was kept constant at 349.15 K.

Once the sample weight stabilized after being degassed for over 10 h, the operating temperature was reduced to the isotherm condition using a Grant water bath. The ionic liquid sample was kept under this condition for at least an hour to ensure that the sample weight was at equilibrium. Once the weight of the sample has been stable, the isotherm process can be initiated by choosing a list of pressures at which the gas solubility is to be measured. Once all the necessary parameters have been selected in the IGA computer software (Version 2010), C_2_H_6_ is injected into the chamber using the mass flow controller (MFC). The system’s pressure is kept constant at a pre-set pressure until the system reaches equilibrium. The IGA-003 records all real-time data, such as weight, temperature, and pressure.

Measurement of the density of the solvent is a required input in the experimental procedure. We reported the values of the densities of the 3 solvents in reference [13]. The density values decreased in the following order: [PMIM][Tf2N] > [HMIM][Tf2N] > [BMIM][DMP]. [BMIM][DMP] was reported to have a high viscosity value of 409.88 mPa·s at 303.15 K [13].

**Figure 11:**
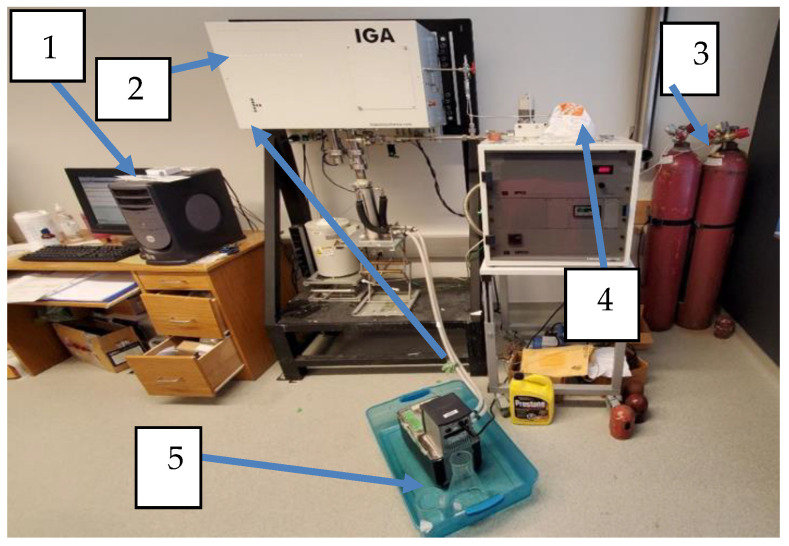
IGA-003 setup.

Figure 11 shows the setup for the IGA. Each instrument used in this work was numerically labelled. The setup consists of (1) a computer, (2) a microbalance, (3) a gas cylinder, (4) mass flow control, and (5) a water bath.

### 4.3. Thermodynamic Modeling

The solubility data of C_2_H_6_ in ILs obtained in this work were correlated using the Peng–Robinson equation of state (PR-EoS), shown in Equation (6) [15], where $a_{m}$ is the intermolecular attractive force and $b_{m}$ is the van der Waals co-volume factor. The PR-EoS combined with three different mixing rules was used to build this model. The three mixing rules are the following:

1. Single binary interaction parameter based on van der Waals one (vdW1);
2. Two binary interaction parameters based on van der Waals two (vdW2);
3. Wong–Sandler mixing rules combined with the NRTL model (WS-NRTL).

(6) $$P=\frac{RT}{v-b_{m}}-\frac{a_{m}(T)}{v\left(v+b_{m}\right)+b_{m}(v-b_{m})}$$

#### 4.3.1. Van der Waals Mixing Rule

For this case, van der Waals created two different mixing rules to estimate mixture parameters ($a_{m}$ and $b_{m}$): van der Waals one (vdW1) and van der Waals two (vdW2) [15,16,17]. In the vdW1 mixing rule, only one parameter (lij) is regressed, whereas, for vdW2, two binary interaction parameters are obtained (lij and kij). The parameter a_m_ for both vdW1 and vdW2 mixing rules was estimated using Equation (7). Using the temperature dependence binary interaction parameters, kij and aij can be estimated with Equation (10). The co-volume factor bm for both vdW1 and vdW2 is estimated with Equation (8) and Equation (9), respectively. Using the interaction parameter (lij), $b_{ij}$ can be estimated with Equation (11).

(7) $$a_{m}=\sum_{i}\sum_{j}x_{i}x_{j}a_{ij}$$

(8) $$b_{m}=\sum_{i}x_{i}b_{i}$$

(9) $$b_{m}=\sum_{i}\sum_{j}x_{i}x_{j}b_{ij}$$

where

(10) $$a_{ij}=\sqrt{a_{ii}a_{jj}}(1-k_{ij})$$

(11) $$b_{ij}=\frac{b_{i}+b_{j}}{2}(1-l_{ij})$$

where $a_{ii}$ and $a_{jj}$ are the pure component intermolecular attractive force parameter and $b_{i}$ and $b_{j}$ are the pure components co-volume factor parameters for the components (i and j).

#### 4.3.2. Wong–Sandler Mixing Rule

The mixture’s attractive force parameter, a, and co-volume parameter, b, can be calculated using Equations (12) and (13) [15], respectively, for Wong–Sandler mixing rules. In this paper, the Non-Random Two-Liquid (NRTL) model is utilized to approximate the activity coefficient and Excess Gibbs Energy (Equations (18)–(24)) [16]. The mixture parameters were estimated using three binary interaction parameters ($k_{ij}$, $\tau_{ij},$ and $\tau_{ji}$), where $\tau_{ij}$ and $\tau_{ji}$ are the NRTL parameters. The value of $\alpha_{ij}$ in Equations (19) and (20) were set at 0.3 arbitrarily in this work. The energies $g_{ij}$ and $g_{jj}$ are the energies of interaction between molecules ‘i’ and ‘j’ [17,18,19] and are calculated using Equations (21) and (22).

(12) $$a=RT\frac{QD}{1-D}$$

(13) $$b=\frac{Q}{1-D}$$

where

(14) $$Q=\sum_{i}\sum_{j}x_{i}x_{j}{\left(b-\frac{a}{RT}\right)}_{ij}$$

(15) $$D=\sum_{i}x_{i}\left(\frac{a_{i}}{b_{i}RT}\right)+\left(\frac{G^{ex}}{CRT}\right)$$

(16) $$C=-\ln\left(\left(1+\sqrt{2}\right)/\sqrt{2}\right)$$

where ${\left(b-\frac{a}{RT}\right)}_{ij}$ in Equation (14) calculated using Equation (17):

(17) $${\left(b-\frac{a}{RT}\right)}_{ij}=\frac{1}{2}\left[\left(b_{i}-\frac{a_{i}}{RT}\right)+\left(b_{j}-\frac{a_{j}}{RT}\right)\right]\left(1-k_{ij}\right)$$

(18) $$\;\frac{G^{ex}}{RT}=x_{i}x_{j}\left(\frac{\tau_{ji}G_{ji}}{x_{i}+x_{j}G_{ji}}+\frac{\tau_{ij}G_{ij}}{x_{j}+x_{i}G_{ij}}\right)$$

(19) $$G_{ij}=exp\left(-\alpha_{ij}\tau_{ij}\right)$$

(20) $$G_{ji}=exp\left(-\alpha_{ij}\tau_{ji}\right)$$

(21) $$\tau_{ij}=\left(g_{ij}-g_{jj}\right)/RT$$

(22) $$\tau_{ji}=\left(g_{ji}-g_{ii}\right)/RT$$

(23) $$\ln\gamma_{i}=x_{j}^{2}\left[\tau_{ji}{\left(\frac{G_{ji}}{x_{i}+x_{j}G_{ji}}\right)}^{2}+\frac{\tau_{ij}G_{ij}}{{\left(x_{j}+x_{i}G_{ij}\right)}^{2}}\right]$$

(24) $$\ln\gamma_{j}=x_{i}^{2}\left[\tau_{ij}{\left(\frac{G_{ij}}{x_{j}+x_{i}G_{ij}}\right)}^{2}+\frac{\tau_{ji}G_{ji}}{{\left(x_{i}+x_{j}G_{ji}\right)}^{2}}\right]$$

### 4.4. Critical Properties Calculations

The critical parameters of the gas and the solvent examined must be known to be used in the model. The modified Lydersen–Joback–Reid group contribution method was used to determine the critical temperature (Tc), critical pressure (Pc), and the acentric factor (ω) of the ionic liquids in this study using the spreadsheet provided by Valderrama and Rojas [20]. Table 8 lists the critical properties determined for this study’s three ionic liquids and C_2_H_6_.

## 5. Conclusions

The purpose of this work was to screen three new ionic liquids, measuring their capacity to absorb C_2_H_6_ at pressures up to 1.4 MPa and at temperatures ranging from 293.15 K to 343.15 K. Thermodynamic properties such as the enthalpies and entropies of absorption are reported and the data used to determine the selectivity values.

The ionic liquids selected were [HMIM][Tf2N], [BMIM][DMP], and [PMIM][Tf2N]. In an earlier publication, we reported that the density values of the solvents decreased in the following order: [PMIM][Tf2N] > [HMIM][Tf2N] > [BMIM][DMP].

As co-absorption of ethane is important in natural gas processing, Henry’s Law constants for C_2_H_6_ were estimated at 303.15 K, 323.15 K, and 343.15 K. As expected, they increased for all ILs as temperature increased. Overall, the ranking of the Henry’s Law constants for C_2_H_6_ is the following: [BMIM][DMP] > [PMIM][Tf2N] > [HMIM][Tf2N] at all temperatures. [BMIM][DMP] is, therefore, found to be the IL that absorbs ethane the least. Compared to other ionic liquids, the three ionic liquids are not the best, but [BMIM][DMP] seems to be the best option. The order in the selectivity can be predicted based on the overall polarity number N and its calculated values obtained from reference [9].

The overall ranking of the CO_2_/C_2_H_6_ selectivity is the following: [PMIM][Tf2N] > [BMIM] [DMP] > [HMIM][Tf2N] > Selexol. The most promising ionic liquid in terms of selectivity is therefore [PMIM][Tf2N]. Note that the three ionic liquids studied had better selectivity than Selexol, a widely used physical solvent in gas sweetening plants.

Solubility data were correlated using the Peng–Robinson equation of state (PR-EoS), and the binary interaction coefficients were reported. The average absolute deviations for the vdW1, vdW2, and WS-NRTL mixing rules when used in the ionic liquids for C_2_H_6_ were 4.39%, 2.45%, and 2.45%, respectively. The enthalpy and entropies of adsorption were calculated. Negative enthalpies were reported, indicating an exothermic process of absorption.

## Figures and Tables

**Table 1:** Solubility of C_2_H_6_ in [HMIM][Tf2N] at 303.15 K, 323.15 K, and 343.15 K.

[HMIM][Tf2N]					
303.15 K		323.15 K		343.15 K	
$x_{C2H6}$	Pressure (MPa)	$x_{C2H6}$	Pressure (MPa)	$x_{C2H6}$	Pressure (MPa)
0.003	0.01991	0.003	0.0199	0.003	0.0199
0.006	0.0499	0.005	0.0499	0.005	0.0499
0.013	0.09989	0.010	0.1000	0.008	0.0999
0.025	0.20004	0.021	0.1998	0.020	0.2002
0.049	0.3998	0.041	0.3997	0.037	0.3998
0.071	0.59988	0.059	0.5998	0.054	0.5998
0.092	0.7999	0.077	0.7998	0.070	0.7995
0.111	0.9994	0.094	0.9996	0.081	0.9999
0.120	1.0998	0.102	1.0998	0.091	1.1000
0.129	1.1988	0.110	1.2011	0.103	1.1994
0.135	1.2495	0.114	1.2503	0.107	1.2513
0.139	1.3007	0.119	1.3009	-	-

Standard uncertainty u(x) = 0.006; standard uncertainty u(T) = 0.1 K; standard uncertainty u(P) = 0.0008 MPa.

**Table 2:** Solubility of C_2_H_6_ in [BMIM][DEP] at 303.15 K, 323.15 K, and 343.15 K.

[BMIM][DMP]					
303.15 K		323.15 K		343.15 K	
$x_{C2H6}$	Pressure (MPa)	$x_{C2H6}$	Pressure (MPa)	$x_{C2H6}$	Pressure (MPa)
0.001	0.01978	0.001	0.0198	0.002	0.0196
0.002	0.0499	0.002	0.0499	0.002	0.0499
0.004	0.09989	0.004	0.0999	0.004	0.1001
0.008	0.19985	0.010	0.1999	0.010	0.1999
0.017	0.39991	0.018	0.4002	0.017	0.3996
0.026	0.59976	0.028	0.6006	0.023	0.5999
0.034	0.79991	0.038	0.7998	0.032	0.7999
0.0422	0.9998	0.045	0.9998	0.043	1.0006
0.050	1.2003	0.054	1.0997	0.047	1.0998
0.056	1.2996	0.059	1.2005	0.053	1.2012
0.059	1.3486	-	-	0.059	1.3011
0.063	1.4006	-	-	0.065	1.4006

Standard uncertainty u(x) = 0.006; standard uncertainty u(T) = 0.1 K; standard uncertainty u(P) = 0.0008 MPa.

**Table 3:** Solubility of C_2_H_6_ in [PMIM][Tf2N] at 303.15K, 323.15K, and 343.15K.

[PMIM][Tf2N]					
303.15 K		323.15 K		343.15 K	
$x_{C2H6}$	Pressure (MPa)	$x_{C2H6}$	Pressure (MPa)	$x_{C2H6}$	Pressure (MPa)
0.002	0.0198	0.003	0.0199	0.002	0.0198
0.005	0.0499	0.005	0.0499	0.002	0.0500
0.009	0.0999	0.008	0.0999	0.005	0.0999
0.016	0.1999	0.015	0.1999	0.011	0.1999
0.032	0.4001	0.029	0.3999	0.023	0.4002
0.045	0.5999	0.042	0.5997	0.033	0.6011
0.058	0.7999	0.053	0.7998	0.044	0.8000
0.070	0.9999	0.065	0.9998	0.054	0.9999
0.083	1.1991	-	-	0.065	1.2012
0.088	1.30132	-	-	0.070	1.2998
-	-	-	-	0.079	1.4000

Standard uncertainty u(x) = 0.006; standard uncertainty u(T) = 0.1 K; standard uncertainty u(P) = 0.0008 MPa.

**Table 4:** Optimized binary interaction parameters for different models and calculated AAD%.

Compound	Temperature (K) and Pressure (MPa)	Mixing Rule	$k_{12}$ ^a^	$l_{12}$ ^b^	$\tau_{12}$	$\tau_{21}$	AAD%
C_2_H_6_(1) + [HMIM][Tf2N] (**2**)	303.15 and 0.1–1.4	vdW1	0.0797	-	-	-	3.19
		vdW2	0.1566	0.0163	-	-	0.47
		WS + NRTL	1.305	-	0.2151	−0.1848	0.44
	323.15 and 0.1–1.4	vdW1	0.0694	-	-	-	2.11
		vdW2	0.1116	0.0104	-	-	0.83
		WS + NRTL	1.2293	-	0.7031	−0.5823	0.84
	343.15 and 0.1–1.4	vdW1	0.0452	-	-	-	3.37
		vdW2	0.0557	0.0026	-	-	3.30
		WS + NRTL	1.0256		0.0396	0.0123	3.30
C_2_H_6_ (1) + [BMIM][DMP] (**2**)	303.15 and 0.1–1.4	vdW1	0.1931	-	-	-	4.77
		vdW2	0.052	−0.0377		-	2.35
		WS + NRTL	0.9997	-	1.1024	0.6362	2.34
	323.15 and 0.1–1.4	vdW1	0.1564	-	-	-	7.13
		vdW2	0.0294	−0.0346	-	-	6.65
		WS + NRTL	1.1384	-	0.6327	0.2915	6.66
	343.15 and 0.1–1.4	vdW1	0.1494	-	-	-	7.40
		vdW2	−0.2053	−0.101	-	-	4.44
		WS + NRTL	−0.1133	-	0.4517	1.8268	4.39
C_2_H_6_ (1) + [PMIM][Tf2N] (**2**)	303.15 and 0.1–1.4	vdW1	0.1240	-	-	-	5.23
		vdW2	0.236	0.0308	-	-	1.47
		WS + NRTL	1.6977	-	−0.0402	−0.0385	1.53
	323.15 and 0.1–1.4	vdW1	0.0987	-	-	-	4.41
		vdW2	0.2757	0.0467	-	-	0.66
		WS + NRTL	1.7468	-	−0.5135	0.1939	0.66
	343.15 and 0.1–1.4	vdW1	0.1028	-	-	-	1.93
		vdW2	0.0678	−0.0093	-	-	1.86
		WS + NRTL	1.1086	-	0.0247	0.3612	1.87

^a^ $k_{12}$ = $k_{21};\;$^b^ $l_{12}$ = $l_{21}$.

**Table 5:** Henry’s Law constants, enthalpies, and entropies for the solvation of C_2_H_6_ in ionic liquids at infinite dilution.

Ionic Liquid	Henry’s Law Constant (MPa)			$∆H^{\infty }$ (KJ/kmol)	$∆S^{\infty }$ (KJ/mol·K)
	T = 303.15 K	T = 323.15 K	T = 343.15 K		
[HMIM][Tf2N]	7.63	9.43	10.69	−1.16	−3.61
[BMIM][DMP]	23.60	22.07	25.93	−0.81	−2.63
[PMIM][Tf2N]	12.85	13.61	17.35	−6.40	−20.02

**Table 6:** CO_2_/C_2_H_6_ selectivity of the ionic liquids used in this work.

	Temperature (K)		
	303.15	323.15	343.15
Solvents			
[HMIM][Tf2N]	2.47	2.32	2.07
[BMIM][DMP]	3.19	2.30	2.01
[PMIM][Tf2N]	3.87	2.98	2.79
Selexol	1.71	1.77	1.88

**Table 7:** List of all the solvents and solutes used in this study, structures, acronyms, suppliers, purities, and water content.

Compounds	Structure	Abbreviation	Suppliers and Purities	Water Content
Ethane	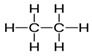	C_2_H_6_	Linde (Regina, SK, Canada) (99.99%)	-
1-Hexyl-3-methylimidazolium bis(trifluormethylsulfonyl)imide	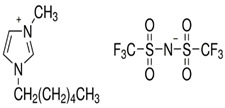	[HMIM][Tf2N]	Sigma-Aldrich (Oakville, ON, Canada) (≥98.0%)	-
1-Butyl-3-methylimidazolium dimethylphosphate	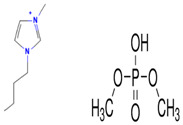	[BMIM][DMP]	IoLiTec (Heilbronn, Germany) (>98.0%)	<2500 ppm
1-Propyl-3-methylimidazolium Bis(trifluoromethylsulfonyl)imide	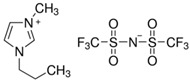	[PMIM][Tf2N]	Sigma-Aldrich (Oakville, ON, Canada) (≥99.0%)	≤0.5 wt%
1-Butyl-3-methylimidazolium hexafluorophosphate	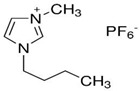	[BMIM][PF6]	Sigma-Aldrich (Oakville, ON, Canada) (≥97.0%)	-

**Table 8:** Molecular weights and critical properties of solutes and solvents used in this study.

Components	MW (g/mol)	$T_{c}$ (K)	$P_{c}$ (MPa)	ω
[HMIM][Tf2N]	447.4	1292.8	2.39	0.3893
[BMIM][DMP]	264.26	851	2.15	0.961
[PMIM][Tf2N]	405.3	1259.3	3.0	0.2575
C_2_H_6_	30.07	305.4	48.8	0.099

## Data Availability

All experimental data acquired are reported in the manuscript.

## Nomenclature

AAD	Average absolute deviation
[BF_4_]	Tetrafluoroborate
[BMIM][DMP]	1-Butyl-3-methyl-imidazolium dimethyl-phosphate
[N1223][FSI]	N-propyl-n-methyl-n,n-dimethyl-ammonium bis(fluorosulfonyl)imide
[C3C1PIP][FSI]	N-propyl-n-methyl-piperidinium bis(fluorosulfonyl)imide
[C3C1Pyrr][FSI]	1-Methyl-1-propylpyrrolidinium bis(fluorosulfonyl)imide
[C4C1Im] [BF4]	1-Butyl-3-methylimidazolium tetrafluoroborate
CO_2_	Carbon dioxide
C_2_H_6_	Ethane
DCN	Dicyanamide
EoS	Equation of state
[FSI]	Bis(fluorosulfonyl)imide
H	Henry’s Law constant
[HMIM][Tf2N]	1-Hexyl-3-methylimidazolium bis(trifluormethylsulfonyl)imide
IGA	Intelligent Gravimetric Analyzer
ILs	Ionic liquids
K	Kelvin
MDEA	Methyldiethanolamine
MEA	Monoethanolamine
P_c_	Critical pressure
PF_6_	Hexafluoro-phosphate
[PMIM][Tf2N]	1-Propyl-3-methylimidazolium bis(trifluoromethyl-sulfonyl)-imide
PR	Peng–Robinson
RSO4	Alkylsulfate
T_b_	Boiling point temperature
T_c_	Critical temperature
vdW1	van der Waals one
vdW2	van der Waals two
WS-NRTL	Wong–Sandler mixing rules + Non-Random Two-Liquid model
x	Liquid mole fraction (moles gas/moles gas + moles solVent)
y	Vapor mole fraction
Z_c_	Critical compressibility factor
